# Judicial Response to Ecological Environment Risk in China—From the Perspective of Social Systems Theory

**DOI:** 10.3390/ijerph192114355

**Published:** 2022-11-02

**Authors:** Tian Sang, Peng Liu, Liang Zhao

**Affiliations:** 1 KoGuan Law School, Shanghai Jiao Tong University, Shanghai 200030, China; 2 Intellectual Property Law and Policy Institute, East China University of Political Science and Law, Shanghai 200042, China; 3 School of Tourism, Hubei University, Wuhan 430000, China

**Keywords:** legal governance, ecological environment risk, risk society, social systems theory, Niklas Luhmann

## Abstract

In modern society, law is one of the most important means of risk prevention and control. Under the challenge of ecological and environmental risks, China’s legal governance experience provides important historical experience and theoretical samples for other countries. Faced with problems, such as the difficulty of eliminating risks, risk decisions themselves bring risks, and the huge social cost of risk response, the social system theory can provide novel and new ideas for the cognition and response of environmental risks. Combining the experience of judicial practice with social theory, especially Niklas Luhmann’s doctrine of the risk/danger dichotomy, a clearer functional orientation can be given to judicial powers based on risk communication and risk attribution. By reviewing the ecological judicial practices in China, Germany, and other countries, the role of the legal system in stabilizing the normative expectations of the whole of society can be summarized, which will provide a reference for the risk response and legal governance of the global ecological environment.

## 1. Introduction

The essence of risk response is to seek as much certainty as possible in a world full of uncertainty. As the most important tool for maintaining the stability and consistency of social expectations, law plays a crucial role in the process of risk prevention and management in modern society. Different from the characteristics of both administrative law and civil law, the field of environmental law has a unique position in the field of risk control. In China’s legal discourse, environmental law is an important part of the “third sector” or “social law area”, which transcends the public/private law dichotomy; its application of rules of rights and obligations will also be more complex. Under the conditions of close interaction between the natural environment and human society, the field of environmental governance involves a series of complex variables, and its calculation sometimes needs to consider the issue of assessment costs. In China today, such realities are particularly evident in the context of a risk society. Faced with new and complex challenges, more complex theoretical tools are often required. The social system theory, which originated in Germany in the 20th century, will provide the main methodology for the argument of this paper. We attempt to summarize the relevant experience in China and apply the framework of social systems theory to focus on the regulation of ecological risks in the context of risk society.

## 2. Challenges of China’s Eco-Environmental Risk Governance in a Complex System

In 2015, The Supreme People’s Court of The People’s Republic of China launched the Interpretation on Several Issues Concerning the Application of Law to the Trial of Environmental Civil Public Interest Litigation Cases, which explicitly includes “Acts of environmental pollution and ecological damage with significant risk of harming social public interests” in the scope of the application of environmental civil public interest litigation (Available online: https://www.mee.gov.cn/zcwj/zcjd/201605/t20160522_343426.shtml, accessed on 14 September 2022, and the full text of China’s newly revised Environmental Protection Law in 2014 is available online: https://www.mee.gov.cn/ywgz/fgbz/fl/201404/t20140425_271040.shtml, accessed on 14 September 2022). It not only provides a clear basis for preventive environmental justice for the first time but also reflects the unique logic of applying such a “the third jurisdiction” from the perspective of public interest litigation [1,2,3] (As a “third field” outside public law and private law, we can look at the evolution of the field of social law in various developed countries since the 20th century. It is guaranteed by the coercive power of the state but does not directly regulate the operation of public power; it deals with the rights and obligations of equal civil subjects based on ecological interests but does not exclusively leave individuals or legal persons as the responsible subjects only. By contrast, traditional public or private law involves only limited factors and can be dealt with through the abstraction of legal relationships).

Accordingly, China has developed a series of studies in the environmental field that make full use of risk society theory. The most representative of which are environmental public interest litigation, energy supply and security, and soil pollution and control. They have a growing body of court precedents, on the one hand, and are associated with relevant scientific expertise on the other. However, compared to the ever-increasing difficulty of governance and the endless number of difficult cases in modern society, there is still pressure to grow theoretically and practically, and traditional judicial strategies are becoming increasingly strained by the superposition of complex factors.

In the final analysis, no modern civilized society can use old-fashioned policy or legal tools to deal with a complex systemic problem without encountering a dilemma that is beyond its capacity. This is reflected in the following three aspects.

### 2.1. Risk Is Something That Can Only Be Prevented, Not Eliminated

The pursuit of a stable order forces people to consider and prevent risks as much as possible, but prevention is not the same as unconditionally pursuing risk minimization. In other words, even the pursuit of “zero risk” can bring about unbearably costly dilemmas [4]. This includes limited technical options, high costs, considerable regulatory resources, high litigation costs, and endless disputes [5]. This brings up the dilemma of defining infringement in the reality of judicial operation, i.e., the cost of assessment and compensation is too high. The current definition adopted in China is to calculate the difference between the existing state of interest and the state of interest that would have occurred if no damage had occurred, and the resulting amount is the scope of compensation.

Guided by the principle of “full compensation”, it requires a combination of multiple forms of appraisal and forms of liability division. So we come to two kinds of amounts, one is the proposed compensation amount, and the other is the actual damage suffered by the ecology. The former should try to match the actual loss of the latter. For example, in the 2015 pollution case in Nanping, Fujian Province, the court sentenced the defendant Xie and others to remove the polluting equipment and waste within five months and restore the function of about 1.9 hectares of damaged woodland; if the removal task and revegetation were not completed on schedule, compensation of CNY 1.119 million was sought for ecological restoration; compensation of CNY 1.27 million was also ordered for the service function during the restoration of the damaged ecological environment losses [6].

When the amount of compensation is huge, the court must face the problem of difficulty in enforcing. For example, in the “7.16 oil pollution case” in Dalian in 2010 [7], the amount of compensation for the recovery of oil pollution alone was CNY 230 million in half a month, not counting the cost of ecological restoration of the entire polluted ocean [8]. Of course, this further reveals the difficulties of the assessment itself, which relies on oceanography, physics, biology, and other disciplines and related scientific and technological tools, and often results in high assessment costs. For example, in the “Tasman Sea” ship oil pollution case [9], the investigation and assessment costs of the Bureau of Oceanography alone were as high as CNY 2.4 million, and the assessment costs of the Fisheries Department were CNY 480,000 [10], which is almost equal to the costs of monitoring, assessment, and research [11]. The risk of loss in the above cases can at least be objectively assessed, while in large environmental tort cases, the loss is likely to be immeasurable, breaking through a specific time frame and having effects for decades or even centuries. Many assessments of ecological catastrophes are beginning to resort to intergenerational equity, in which case traditional rules of compensation may not apply. These complicating factors are what the legal system has to consider.

### 2.2. The Decision of Risk Treatment May Lead to Risk

A typical paradox in the field of risk regulation is that the process of reducing one risk may bring another new risk. In risk society theory, this is known as additional and alternative risks. For example, the safety of nuclear power plants, which is often discussed in European countries, is difficult to avoid the resource pressure of fossil energy combustion by preventing the construction of nuclear power plants under the condition that human energy demand is only increasing, and thermal power generation is, at the same time, one of the important factors causing global climate change and acid rain problems. Another example is the global ban of DDT [12,13], which has aggravated the spread of dysentery in less developed countries [14]. Currently, countries respond to environmental risks by relying mainly on risk assessment and risk decision-making instruments, such as the valuation of damages and their compensation in environmental tort cases. This approach presupposes the existence of an objective object that can be recognized and evaluated and a complete risk criterion that can be predetermined, measured for each specific environmental case, and then given a complete or even the only correct judgment. However, such a “holistic approach” is not feasible in the operation of real society, and the multi-disciplinary linkages of modern society do not satisfy linear causality.

Therefore, the duty of risk prevention does not mean the uncontrolled reduction in a particular risk in a certain area, which is directly reflected in jurisprudence by the question of determining the “residual risk”. The term “residual risk” refers to a risk that cannot be eliminated in principle and should be endured by society. This concept originated in the Kalkar Case of the Federal Constitutional Court of Germany in 1978. According to the decision of the Kalkar case, the use of nuclear energy is limited by human perceptions, absolute safety is not desirable, and the whole of society must bear the “residual risk” [15,16] (in the field of epidemiology, this concept also has important inspiration. Now more and more politicians and scholars have noticed it. This is a phenomenon unique to modern society) of nuclear energy development [17,18]. German jurisprudence has, thus, developed a three-part theory of “danger, risk, and residual risk,” in which risk is probabilistic but can be held accountable, while residual risk is exempt, according to society’s standard of practical rationality; in contrast, danger is the more traditional counterpart of tort liability. In contrast with this, the state is obliged to pay attention to, intervene in, and exclude the risk; the treatment of the risk requires additional technical possibilities, proportionality between the means of intervention and the benefits achieved, and the judiciary can make a moderate judicial review of some of the risky decisions; the “residual risk” cannot be excluded in principle and belongs to the scope of the obligation of all. In principle, the “residual risk” cannot be excluded, and it is within the scope of the obligation of all citizens to tolerate it, which, of course, also reserves the space for risk communication, risk attribution, and risk sharing based on public negotiations. Thus, by means of a legal fiction and typology, Germany has essentially adapted to the ecological risks of modern society through the balancing of interests (in addition, environmental risk governance in other East Asian countries is also worthy of attention. For an overview of this topic, see Adeel [19]).

### 2.3. The Unknown and Uncertainty Always Accompany the Risk Assessment

Modern society is a complex society, in systems theory terms, it is a complex system based on multiple causes and effects. The reason for the emergence of risks in modern society is that human beings are full of unknowns about the future, and even “don’t realize that they don’t know”, which means there is no way to predict what may happen and the linkage between things. Especially for legal norms, it is necessary to make future-oriented decisions based on existing experience. Legislators and even judges should base their decisions on the present and plan for the future. We can also call it embedding the “future present” into the “past present”. However, it is obvious that the act of overriding or displacing previous experience to extrapolate what may happen in the future is itself leading to uncertainty. Such assessments and calculations have not got practical significance compared to the growing problem of ecological risk. For example, in China today, there is no comprehensive natural resource asset system, and it is difficult to accurately measure ecological damage, and even if a national natural resource balance sheet is prepared, it is theoretically impossible to include ecological values that cannot be measured.

New theories are often needed to deal with situations that have never been seen before in history, as are tools to deal with complex problems that have never been seen before. Here, it is necessary for us to submit the issues of law to the broader social sciences, introducing new thought resources to understand the challenges of environmental governance and solve a series of concrete problems, such as “How to respond to ecological risks” [20,21]. At present, China has paid close attention to and deeply studied some important Western risk society theories and applied these “Risk” theory tools to the specific environmental governance analysis. One of the most representative, for example, is the work of Ulrich Berk [22,23] and Anthony Giddens [24,25,26]. In recent years, however, it has been the German sociologist Niklas Luhmann’s theory of the risk society (of course, many other European and American jurists and sociologists also have some important explorations, such as Taylor-Gooby and Zinn [27] and Cass [28]) that has had the greatest impact on the Chinese sociology of law [29,30,31,32].

Luhmann is known for his tools of “social systems theory”. The systems-based theory of risk, which is also an important theoretical legacy of twentieth-century sociology, is a second-order theory that examines the question of “how”, that is, it puts aside the question of “what” risk itself is as an object, and thus examines how the system itself observes and responds to risk, that is, observing the observation. On this basis, Luhmann suggests that “risk/safety” is not a valid set of distinctions, and that what is really revealing is “risk/danger” distinction, which could open up new horizons for understanding ecological risk regulation.

## 3. Risk Control Techniques Based on the Risk/Danger Dichotomy

In constructivist social system theory, “risk” is not an objective entity, but a socially constructed phenomenon. In traditional societies, uncertainty itself was perceived but not equated with the modern concept of “risk”. In the classical era of the major axial civilizations, for example, the treatment of uncertainty often resorted to fate or moral qualities, such as attributing favorable or unfavorable outcomes to “Fortuna” or requiring decision-makers to remain “Prudentia”. This is, in fact, the practice of transferring responsibility for actions to the causal chain, finding a subject of attribution that can be “suspended”, or resorting to mysterious forces beyond the power of man for unfavorable outcomes. This is reflected in the etymology of the word “risk”. In Italian, “risk” was first associated with navigation, its original meaning is to tear, and the directly related image is that of the reef or the rock, which refers to the clear and probable danger that a cargo ship will encounter during a long voyage. However, the word “risk” here mostly refers to natural and objective phenomena, such as natural disasters on the sea; but with the development of technology and the deepening of human knowledge and control of natural phenomena, fewer and fewer people entrust risk control to external natural conditions or mysterious forces but are increasingly linked to personal decisions [29].

### 3.1. Reconceptualizing the Counterpart of Risk in Modern Society: Not “Security”, but “Danger”

In the old European conceptual world, the idea of appealing to external forces or personal qualities could still meet the challenge of the “simple society”, but it was a rather rudimentary approach to complexity reduction, which could only face and deal with very limited uncertainties. The social order of human beings originates from the selection of reusable structures from the infinite complexity; as the complexity of society increases, it becomes more and more difficult to maintain order, and society itself moves towards a stage of functional differentiation, i.e., a series of functional sub-systems evolve to deal with various specific problems. These sub-systems tend to have closed boundaries, for example, the political system focuses on political issues according to “authority/non-authority”, while the legal system concentrates on legal matters according to “legal/illegal”. Only in certain circumstances does the linkage between systems occur. As a representative example, the Constitution is the point of contact that couples the political and legal systems (Luhmann expounded this point of view in different works, the most representative book is Luhmann [33]. In addition, constitutional scholars in the United States have also paid special attention to the relationship between risk and the constitution, although this does not necessarily require the use of the theoretical tools of systems theory. See Vermeule [34] and Sunstein [28]). Each system takes the output of the other system as its own input, and, at the same time, operates according to its own logic to produce further output.

With the advent of modern society, the expansion of the market economy has made efficiency the most important value goal, and the “externalities” it brings—such as the environmental issues to be discussed here—become a by-product that puts pressure on other systems. The output of one system becomes the input of another system. This pressure from the economy requires political and legal responses, for example, to improve social welfare and ecological protection through policies or legislation, so as to make up for the externality of ecological damage caused by the economy. However, systems are related to each other. If each system deals with this “externality” of other systems, the complexity generated by the superposition will accumulate sharply, and the uncertainty will multiply. Such phenomena mutually reinforce the process of functional differentiation in modern society. In other words, this historical process is the inevitable direction of modern society, and its rationale faces and deals with the multiplicative complexity from the environment by strengthening its own complexity.

According to Luhmann, the opposite of “risk” in modern society is not “safety” but “danger”. In fact, “safety” is not a real and valid concept, and there is no measurable safety in the absolute sense, but only “no risk”. Whereas, on the contrary, risk can be quantified. In contrast, a more valid distinction is made between risk and danger, which involves adverse consequences based on the decision of the responsible person, sometimes a risky choice made after a thorough evaluation, and danger, which cannot be attributed to the decision maker but is the result of an external event [35,36,37,38,39,40]. This makes a further explicit refinement with the aforementioned trichotomous framework of danger, risk, and residual risk, adding the dimension of subjective decision making to the perception of risk. The inspiration from this new distinction can be used not only to judge the subjective fault of suspects in criminal law but also to measure the level of risky decision making of decision makers, which has a wide scope of application in issues involving public interest, such as environmental law [41,42]. All the above is a “translation” of the previous part of the dilemma and challenge, which is based on the functional analysis of social systems theory. Through such a new approach, we could find the response to ecological risks is at a crossroads.

### 3.2. The Paradox of Risk Control and Risk Decision in Modern Social Systems

Based on the dichotomy between danger and risk, it is necessary for society to incorporate the reflexivity of risk into its own observation and operations, that is, to recognize the contingent and linked nature of the mechanisms of risk occurrence, and thus to enhance its ability to cope with environmental complexity. From the position of second-order observation, the social system is not so much addressing ecological risk directly as it is attempting to distribute the consequences of risk within society after recognizing that risk cannot be eliminated. Here, social systems theory is unique in stating that the basic unit of operation of social systems is “Kommuninkation”, rather than events, rules, or even social organization, as we usually understand them [33] (for example, the legal system is constantly maintained through “legal/illegal” continuous communication, we have mentioned this above, and Luhmann has an excellent discussion on this in his monograph of the legal system). In social systems theory, the “system/environment” distinction is a dominant Leitdifferenz, and the functioning and survival of all systems depends on the continued validity of this distinction, i.e., the drawing of a boundary between the system and the environment. On this basis, the maintenance of the boundary depends on a set of recursive self-reproduction mechanisms, which laid the theoretical foundation for Luhmann’s monograph on ecology and legal governance—Ecological Communication.

Specifically, modern societies have evolved a unique capacity for risk dilution—a dilution in which the consequences of risk are shifted or forgotten through specific social means—and the political system plays a prominent function here. In the face of risk, the political system is responsible for deflecting or redistributing social problems, but it does not seek to eliminate risk itself. It has some amount of ability to shift issues so that regulatory failures are quickly forgotten, and, for this reason, post-event apologies for regulatory failures become the norm for the political system to respond to society. The political system’s decision to control and plan for technological and environmental risks is policy. When people invent and use technologies, there are always causal relationships that cannot be incorporated into the technological horizon. Therefore, there is always the possibility of risk residuals that cannot be planned and controlled in advance. At the same time, the political system itself becomes a potential source of triggering and even amplifying risks.

More seriously, non-decision making [43] or late decision making is itself a form of decision making, such as not dealing with a nuclear leak when it occurs. Making decisions, avoiding decisions, or postponing decisions may lead to unanticipated risks, and such risks need to be attributed to those responsible, which is very different from disasters of a purely natural nature [44]. This functional orientation of the political system deconstructs the classical liberal understanding of the political process as mutually supportive of individual freedom and a contractual system based on free will between subjects. According to this traditional understanding, third parties who are harmed without consent have access to remedies through judicial channels. However, the interface between liberty and contract is severed when modern society has seen the dichotomy of risk and danger. Risks resulting from decisions that can lead to large-scale catastrophes, the damaging consequences that can no longer be absorbed by the ex-ante arrangement of rights and obligations by contract, nor can they be compensated for by ex-post tort damages. Ecological risks are the best example of this scenario.

This reality has also brought about two by-products: on the one hand, the state has assumed a clearer and more necessary public obligation. The intervention of the state in civil society is no longer limited to the guarantee of individual rights and free markets, but the prevention of potential environmental risks and the provision of general security based on scientific and administrative means have become important foundations for ensuring the legitimacy and effectiveness of power. Unlike private risks, public risks are not under the control of individuals and can hardly be attributed to the fault factors of specific responsible parties. This requires the state to integrate scientific, economic, and political conditions and make measurements in a holistic perspective. On the other hand, the function of the legal system to maintain social stability expectations has been more emphasized, and judicial power is no longer understood as an entirely passive power; the socially responsive nature of environmental justice is increasingly reinforced. For example, in the 2014 environmental infringement case in Taizhou, China, six defendants were ordered to pay a total of CNY 160,666,745.11 [45] in compensation for environmental remediation costs, of which Jiangsu Changlong Agrochemical Co., Ltd., alone was responsible for about CNY 80 million [46]. This seems to be a very typical case with clear facts and clear tort liability. However, due to the simplification of legal provisions and judicial procedures, it is difficult to implement this decision and more difficult to produce demonstration effects in the social field. In order to remedy this dilemma, the second instance judgment of the case added an amendment: providing that the environmental risk was significantly reduced, the defendant could receive a 40% credit for the funds invested in the technical renovation if it was not punished again within one year for the environmental violation [47]. In this case, the court took the initiative to go beyond the legal provisions and interpreted the relevant legal provisions in favor of the implementation of the environmental policy, which is a very typical result of active social regulation. We will discuss this further in this paper.

### 3.3. Constructivist Attribution and Blame Techniques

At the root, risks will always exist and will cascade if decisions about risk are made continuously. Faced with a situation in which extreme risks may arise at any moment, modern society has developed unique social technologies. Technology is often thought of as the means by which people qualify cause-and-effect relationships through scientific knowledge or practical experience for certain purposes. The so-called “social technology” is essentially one of these broad qualifying devices, which selects factors in an infinitely complex world to form solid connections that are ready to be “called upon” and can be constantly strengthened or improved. It is only that social technology, as opposed to broader technology, is based on social communication [48], that is, the formation of a set of communication patterns that can be used repeatedly to distribute predictable favorable or unfavorable consequences among people and organizations, and it is, therefore, necessarily constructivist. Conversely, if one does not realize that technology is constructivist, they would be trapped in an infinite chain of causal interrelationships.

Technology is a simplification of causality, and its central aspect lies in attribution. Risk events that may occur in the future are different, but the pattern of relationships is already stored and reproducible. Therefore, the social system is built with stable connections between factors that can cope with “similar but different” situations. Or even if deviations occur, they can be studied and responded to “without surprises” through a pre-complicated institutional apparatus that leaves room for discretion in adapting to the situation. Thus, the essence of attribution is to select/exclude factors—to select certain factors as “causes” and potentially discarding others— and thus an already potentially subjective division of responsibility is produced. (In the field of criminal law and tort law, the theory directly related to this is the “proximate cause”, which emphasizes that the objective causality is unlimited and makes choices in the infinitely complex world causality chain, so that specific phenomena can be recognized and handled. For a theoretical overview of this, see Zou [49].) For example, legal economics tends to place efficiency above ethical considerations when it comes to attributing ecological risk and responsibility [50,51,52]. When there is a huge cost to assess, it becomes unwise to clarify the phenomenon at all costs based on social costs and problem solving. Different disciplines and even different schools of thought offer very different ways of attributing risk and responsibility, and ecological legislation and justice in various countries have also weighed in on this, but often as a result of a combination of constructs that are different from both legal economics and purely ethical considerations.

The process of attribution indirectly brings about the process of liability allocation, which together constitute the core aspect of risk decision making, which further constitutes the most basic premise of risk control. In June 2016, China’s Ministry of Environmental Protection had issued the Technical Guidelines for Identification and Assessment of Eco-environmental Damage General Program and the Ecological Environmental Damage Appraisal and Assessment Technical Guidelines Damage Investigation and subsequently carried out a pilot reform of the ecological environment damage compensation system in seven provinces. In the national-level Outline of the 13th Five-Year Plan for National Ecological Protection, issued in October 2016, China further clarified the promotion of the establishment of a sound system for the development and protection of national land space, as well as a comprehensive system for ecological and environmental damage assessment and compensation and ecological protection compensation. This series of documents extracts specific factors from the complex chain of causality or correlation and gives clear criteria and means of traceability, so that future ecological and environmental damage becomes “manageable” in advance [53].

The future is always unpredictable, but as the most important way of expectation management, legal norms provide a mimetic way of “guiding future uncertainties in advance”, even if the present measurement of interests is likely to be contrary to, or at least incompatible with, the future situation, but in the face of the complexity of infinite variables [31]. The normative guidance is better than a blank slate. Thus, this formulation of the legal system is still the result of a subjective distribution and trade-off, which, in fact, reduces the complexity of the passage from the present to the future. In the following, taking the “precautionary principle” as a keyword, we will focus on the legal system, especially the judicial system under the premise of ecological risk regulation (Luhmann conducted his research on the legal system based on the theory of anticipation. In addition to his research on the sociology of law, his main research also includes research on trust issues, see Luhmann [54]).

## 4. The Flip Requirement of the Risk Prevention Principle for the Judicial System: From Reactive to Relatively Proactive

The “precautionary principle” originated in the Vorsorge Code of the Federal Republic of Germany, a term that means “prior concern and consideration” in German. As a principle in the field of environmental law, its core requirement is to prevent potentially harmful actions from damaging the ecological environment through forward planning, resulting in a paradigm shift from “environmental allocation law” to “environmental protection law”. The Swedish Environmental Protection Act of 1969 explicitly invoked the general precautionary principle, but what really distilled it as a legal principle was Germany’s environmental policy, notably the Clean Air Act of 1976. In 1970, the U.S. Congress enacted the Clean Air Act (Draft), which also explicitly used the concept of “risk prevention”. Until the Second International North Sea Conservation Conference in 1984, the German government proposed this principle as an initiative, which was adopted and recorded in the London Declaration [55,56,57]. Its central interpretation is that “the precautionary approach is necessary to prevent possible damage from hazardous substances in the North Sea, and it can require action to control the discharge of such substances even in the absence of clear scientific evidence that proves a causal relationship” [58]. Since then, the precautionary principle of risk has often been emphasized in international environmental protection documents from the 1990s onwards.

Clearly, in this principle, we can see that “even in the absence of a causal judgment based explicitly on scientific research”, this principle requires proactive regulation. In 1992, the Rio Declaration on Environment and Development, adopted by the United Nations Conference on Environment and Development, further distinguished between “prevention” of risk and things that “prohibit” risk, which applies to risks that have already been quantified, and “prevention”, which may be the result of uncertainty about the mechanisms of causation and even the probability of occurrence. The latter applies to risks that have already been quantified, while the former may be the case where the mechanism of causation and even the chance of occurrence are not fully determined. This has also had a clear impact on Chinese legislation, as China passed the Law of People’s Republic of China on Environmental Impact Assessment in October 2002, which states in Article 1: “This Law is formulated for the purpose of implement sustainable development strategies, preventing adverse impact on the environment due to execution of plans and construction projects, and facilitating the coordinated development of the economy, society and environment”. For the first time, emphasis was placed on the prediction, advance analysis, and evaluation of the environmental impact before project implementation. It clearly emphasizes measures to prevent or mitigate negative effects. Later, China’s administrative regulations also began to introduce prior approval procedures from the field of hazardous chemicals and agricultural genetic modification through the institutional device of administrative permits to make a review in advance and avoid possible damages in advance, even if the chances of such damages are highly capricious (in addition to China, other developing countries also have similar explorations; there is some recent literature, such as Rajamani [59] and Hilbeck et al. [60]). By 2019, China’s legislative document, the Soil Pollution Prevention and Control Law, has 125 “risks” and a special chapter IV on “Risk Management and Control and Remediation”, which provide clear and feasible measures to regulate risks to the soil environment [61]. Thus, we can try to distill the core nature and requirements of the prevention principle.

First, it has a distinct public interest, the tort subject and the interested party’s recourse is relatively vague, and the legislation cannot set all the clear rights and obligations norms in advance, and thus prescribe all the legal relations that may emerge. Such norms are slightly generalized under the title of “environmental rights” and, therefore, can only be relaxed in the judicial process to reserve space for environmental public interest litigation. Obviously, such a form of adjudication is completely unthinkable in an era of sharp separation between public and private law. In the face of complex ecological risks, (Luhmann once discussed this from the perspective of social complexity and social continuity, see Luhmann [62] and Valentinov [63]) the threshold for judicial intervention is lowered: if there is a possibility of harm, the institutional presupposition of “harm is actually occurring” can be breached and a positive judicial response can be made. As a result, the judicial power also requires the ability to regulate environmental risks.

Second, it is explicitly based on the condition of the uncertainty and unknowability of the future [64,65] (in the field of law and economics, risk was once understood through cost and probability; as a research field that intersects natural sciences and social sciences, the response to climate change risks is also a typical field to deal with uncertainty). which is directly related to the complex situation of modern society described above, especially in the case where the legislator “knows that he does not know” [66] or even “does not aware that he does not know”, and it is impossible to foresee all the exceptionally difficult situations in the future. However, legal norms should account for this in advance, providing a legal basis for judicial decisions and stable expectations for society. In recent decades, with the rapid growth of China’s economy and the increasing pressure on environmental protection, many Chinese cities have experienced NIMBY, which, in turn, has led Chinese officials to focus on the scientific assessment of risks and the allocation of responsibilities [67]. Just like developed countries in the West, China is increasingly recognizing that environmental risk is not only an objective phenomenon but also exists in the subjective perceptions of the public and that the psychological anxiety and dissatisfaction that permeate the public can accumulate into a wider range of secondary social risks. This has led to a gradual shift in the judicial system from passive post-event regulation to an act.

Therefore, the precautionary principle requires joint action of administration and justice to transform from passive infringement compensation to active risk prevention. Even the judiciary itself must assume the function of implementing policies and education to achieve forward-looking environmental risk management (The forward-looking risk prevention and control in the field of climate change is a good example, just as Nordbeck et al. [68] and Palea et al. [69]). Since it is forward looking, it necessarily requires a pre-determined “interest balance” (for the sake of this measure of interest, we can claim that all prior protection is a social protection, not a natural event, see Holzmann [70]). In particular, in the context of China’s growing demand for a market economy, a proactive judicial power needs to anticipate the government’s policy preferences. Sometimes it can even oscillate with the changing focus of conscious policy, which itself is time-sensitive, i.e., it tends to have a different focus at different stages, such as efficiency, equity, energy security, citizen health, etc. In this case, the judiciary needs to apply the established legislative texts based on the textual interpretation, and at the same time to adapt and interpret the focus of the national policy, and environmental risks become one of the multiple factors to be considered.

These three new features of public interest, the unknown, and the forward looking have led to a flip in the functional orientation of the judicial system, allowing the previously passive and reactive judicial power to begin to take a proactive turn. This shift does not increase the security of society, but it does provide a set of institutionalized coping mechanisms. Risks can be shifted through the attribution and division of responsibility but can never be eliminated. The more complex modern society becomes, the more this fact of impossibility of elimination is reinforced (for a more detailed introduction to Luhmann’s social theory through political and legal theory, see King and Thornhill [71]). “The precautionary principle, developed within the legal system, functions not to increase the level of security in society, but as a procedural response mechanism to absorb the environmental complexity due to the uncertainty of scientific consequences” [72]. Whereas the traditional approach to the measurement of interests also requires prior consideration, legal norms can hardly provide antecedent guidelines for all future actions in unknown situations. Not only is it difficult to give ready-made answers based on legal relationships and patterns of behavior but it is also even difficult to figure out what the impending risk itself is. Therefore, the traditional logic of tort litigation often only provides for such means as the injunction and preservation of conduct before the proceedings, as well as cessation of infringement, removal of obstruction, and elimination of danger during the proceedings; it is almost beyond the reach of the field of risk attribution, risk decision making and risk communication required by the principle of risk prevention.

This situation has improved with the introduction of China’s own Civil Code in 2020. In the tort liability section, the Chinese Civil Code specifically places preventive liability in the general provisions, echoing it with the principle of imputation of damages, i.e., giving equal weight to ex-post liability and ex-ante preventive liability. This provides a preliminary and comprehensive normative basis for the interpretation and application of existing laws to solve the problem even if a new type of ecological risk threatens [73]. In the field of environmental tort and ecological risk prevention, this set of solutions can be developed as follows: (1) to exclude existing and emerging environmental damage; (2) to exclude or mitigate possible or potential environmental risks in the present; and (3) to prevent future environmental harm through the adoption of preventive measures. This allows society to construct a stable expectation of “manageable risk” and to maintain continuous communication with society so that it does not come to a standstill due to sudden or major disasters. Thus, while such norms do not increase the security of society, they provide an institutionalized coping mechanism and make the legal system highly sensitive to changes in other social sub-systems, such as science, politics, and economics.

## 5. Conclusions

Based on the law to maintain the stable normative expectations of the whole of society, the whole of society can communicate based on the principle of fairness, in terms of the perception and assumption of risks. In essence, since risk cannot be eliminated but only distributed, the control of risk relies especially on effective communication among multiple subjects, especially through substantive participation in projects involving their own interests, retaining their right to know and claim, forming a social consensus based on risk communication, and raising the threshold of risk tolerance [62]. On the other hand, we need to respond to the changes of the times and emphasize the changes of judicial power. In principle, of course, it still must abide by the passive position, but it is possible to consider both social expectations and the framework of legislation, through the interpretation and application of the provisions to achieve risk communication. By the understanding of the “third jurisdiction”, we will find it is neither a passive adjudication between the two sides of the defense, nor an intervention of power in the ex officio model, but an argumentative process with stakeholder communication under the auspices of a judge. When residual risk is necessary, negotiation based on the right to know and the principle of fairness can also provide a due process for assigning responsibility [74].

If we compare the environmental governance experience of Western countries since the 1960s with that of China, we will find that in many areas, the exploration made in Europe and the United States is prescient, and these valuable experiences can also constitute a guide or a survey for China. In particular, social systems theory provides a powerful analytical force for ecological risk regulation in modern complex societies. The risk society theory based on social systems theory is equivalent to a screwdriver in a whole toolbox, where it simultaneously works closely with other tools to form a unique theoretical system that tries to observe modern complex societies. This will undoubtedly be of great benefit to all countries in the world as they work together to deal with known and unknown ecological risks.

## Data Availability

Not applicable.
